# Editor’s Review of Key Research Papers Published in *Tomography* during the Last Year

**DOI:** 10.3390/tomography9020069

**Published:** 2023-04-13

**Authors:** Emilio Quaia

**Affiliations:** Department of Radiology, University of Padova, Via Giustiniani 2, 35128 Padova, Italy; emilio.quaia@unipd.it; Tel.: +39-049-8212375

*Tomography* is an open access journal dedicated to all aspects of imaging science from basic research to clinical applications and imaging trials. As Editor-in-Chief of *Tomography* it is my great pleasure to provide a summary of some of the most cited and viewed publications in *Tomography* in 2021–2022 to summarize the last year’s most relevant discoveries in clinical imaging.

Presently, artificial intelligence (AI) and patient radiation exposure likely represent the most relevant general research fields in clinical imaging. Several papers were published last year in *Tomography* regarding AI and, in particular, in AI oncologic imaging applications. Takahashi et al. [1] showed how deep learning (DL) can classify maximum intensity projection (MIP) images from PET-CT data as positive or negative for cancer. DL could also be used to correctly classify breast lesions detected on an X-ray mammography to reduce the false positive recall rate and improve the efficacy of breast cancer screening [2]. Park et al. [3] showed that radiomics features of ductal carcinoma in situ on breast MR imaging may predict ipsilateral tumoral recurrence, while DL-accelerated MR imaging may improve image quality in MR images of musculoskeletal tumors [4]. AI could also be implemented in imaging reconstruction and automatic diagnoses to reduce radiologist workloads. DL can provide high resolution 3D images from native 2D images by using active data interpolation to produce super-resolution high quality MR images [5]. The use of DL to recognize COVID-19 lung disease represents a relevant hot topic that recently garnered a high amount of interest. Yang et al. showed that fast and automatic recognition of COVID-19 disease is possible using different DL algorithms [6], which can relieve the stress of radiologists in screening for COVID-19 infections.

Regarding occupational radiation protection, specific attention has been recently dedicated to the eye lens. The International Commission on Radiological Protection (ICRP) adopted the new recommendation of reducing the occupational eye lens dose limit from 150 mSv/year down to 20 mSv/year averaged over 5 years since cataracts can occur at lower radiation doses than those examined in previous epidemiological research. In a recent paper published in *Tomography*, Inaba et al. [7] showed how neck dosimeters underestimate the eye dose during CT fluoroscopy by approximately two-fold suggesting the use of a direct eye dosimeter is required to accurately measure the eye lens dose.

COVID-19-related pneumonia still represents a main research topic in the radiological literature. In particular, imaging findings in long-COVID still represent a relevant field. Besutti et al. [8] showed that most survivors after severe COVID-19 pneumonia revealed normal chest CT findings, whereas non-fibrotic changes—including non-fibrotic non-specific interstitial pneumonia (NSIP), ground glass changes, and/or organizing pneumonia—in 37% of patients, fibrotic changes—including fibrotic NSIP pattern with subpleural reticulations, traction bronchiectasis, and ground glass changes—in 4% of patients, and post-ventilatory changes—cicatricial emphysema and bronchiectasis in the anterior regions of the upper lung lobes—in 2.5% of patients were identified 5–7 months after severe pneumonia. Baratella et al. [9] demonstrated that digital tomosynthesis presents a higher diagnostic accuracy compared to chest X-ray in revealing fibrotic changes in patients who have recovered from COVID-19 pneumonia and could represent an alternative imaging tool for patient follow-up after COVID-19 pulmonary infections. Finally, Corsi et al. [10] showed that CT structural abnormalities may persist in most COVID-19 survivors despite normal pulmonary function tests.

In conclusion, *Tomography* still represents an important venue for radiology research especially in the most relevant topics of imaging science.
